# Renal Doppler Resistive Index as a Marker of Oxygen Supply and Demand Mismatch in Postoperative Cardiac Surgery Patients

**DOI:** 10.1155/2015/763940

**Published:** 2015-10-29

**Authors:** Francesco Corradi, Claudia Brusasco, Francesco Paparo, Tullio Manca, Gregorio Santori, Filippo Benassi, Alberto Molardi, Alan Gallingani, Andrea Ramelli, Tiziano Gherli, Antonella Vezzani

**Affiliations:** ^1^Anaesthesia and Intensive Care Unit, E. O. Ospedali Galliera, 16128 Genoa, Italy; ^2^Department of Surgery, University Hospital of Parma, 43100 Parma, Italy; ^3^Department of Internal Medicine and Medical Specialties, University of Genoa, 16132 Genoa, Italy; ^4^Radiology Department, E. O. Ospedali Galliera, 16128 Genoa, Italy; ^5^Department of Surgical Sciences and Integrated Diagnostics, University of Genoa, 16132 Genoa, Italy

## Abstract

*Background and Objective*. Renal Doppler resistive index (RDRI) is a noninvasive index considered to reflect renal vascular perfusion. The aim of this study was to identify the independent hemodynamic determinants of RDRI in mechanically ventilated patients after cardiac surgery. *Methods*. RDRI was determined in 61 patients by color and pulse Doppler ultrasonography of the interlobar renal arteries. Intermittent thermodilution cardiac output measurements were obtained and blood samples taken from the tip of pulmonary artery catheter to measure hemodynamics and mixed venous oxygen saturation (SvO_2_). *Results*. By univariate analysis, RDRI was significantly correlated with SvO_2_, oxygen extraction ratio, left ventricular stroke work index, and cardiac index, but not heart rate, central venous pressure, mean artery pressure, pulmonary capillary wedge pressure, systemic vascular resistance index, oxygen delivery index, oxygen consumption index, arterial lactate concentration, and age. However, by multivariate analysis RDRI was significantly correlated with SvO_2_ only. *Conclusions*. The present data suggests that, in mechanically ventilated patients after cardiac surgery, RDRI increases proportionally to the decrease in SvO_2_, thus reflecting an early vascular response to tissue hypoxia.

## 1. Introduction

In humans and animals, various quantitative and semiquantitative Doppler parameters have been proposed to quantify renal blood flow. Among these, renal Doppler resistive index (RDRI) measured from intrarenal arteries is the one most widely used for clinical investigations since it does not require estimations of Doppler angle or vessel cross-sectional area [1]. Moreover, animal studies have shown that RDRI is dependent on perfusion pressure [2] and is increased by hypotension in the presence of hypovolemic or normovolemic anemia [3].

During low-flow states, splanchnic hypoperfusion and blood flow-redistribution are part of the physiological response to oxygen supply and demand mismatch. In two previous studies, RDRI has been shown to be able to detect tissue hypoperfusion and oxygenation due to occult hemorrhagic shock in hemodynamically stable polytrauma patients [4] and to correlate with levels of arterial standard base excess and expression of tissue hypoxia [5]. Moreover, in patients with acute respiratory distress syndrome, high RDRI values were related to mild hypoxemia due to short-term low fraction of inspired oxygen (FiO_2_) [6]. Based on the above findings, it can be hypothesized that changes in RDRI reflect a vascular response to tissue hypoxia in postoperative cardiac surgery patients with oxygen supply and demand mismatch.

This hypothesis was tested in the present study by searching for independent hemodynamic correlates of RDRI in mechanically ventilated patients after cardiac surgery.

## 2. Material and Methods

### 2.1. Patients

The study was approved by institutional review board of our university hospital (protocol number 812/2014). Study protocol and aim were explained to patients before surgery and each of them gave a written informed consent.

Sixty-one consecutive patients admitted to the intensive care unit after elective cardiac surgery were included in the study (Table 1). They were required to have a pulmonary artery catheter in place as per clinical indications and satisfy the following inclusion criteria: (1) age > 18 years; (2) absence of acute kidney injury or ongoing recovery from acute kidney injury; (3) no history of chronic renal failure; (4) absence of any condition known to modify renal Doppler resistive index, namely, suspected or confirmed obstructive renal failure, arrhythmia, renal artery stenosis, sepsis, mitral or tricuspid regurgitation, and intra-abdominal hypertension; (5) no renal replacement therapy; (6) no conditions making RDRI examination not reliable; and (7) no conditions needing mechanical ventilation with positive end-expiratory pressure > 5 cm H_2_O or FiO_2_ > 50%.

All patients were mechanically ventilated with tidal volume of 8–10 mL/kg of predicted body weight and positive end-expiratory pressure of 5 cm H_2_O and FiO_2_ 50%. They were sedated by continuously infused propofol. At the time of inclusion, no patient was receiving renal replacement therapy or had overt acute renal failure. RDRI measurements were performed immediately before hemodynamic measurements, at admission in intensive care unit (ICU), and within the first 12 hrs whenever needed per clinical condition.

### 2.2. Measurements

#### 2.2.1. Hemodynamic Monitoring

Patients were positioned 30° supine and all pressure transducers were referred to mid chest at the level of right atrium. All patients had a radial arterial catheter (*Arterial Leadercath 3F*,* Vygon*,* Ecouven, France*) and a pulmonary artery catheter (*141HF7*,* Edwards Lifesciences, Unterschleißheim*,* Germany*). Clinical data were collected from the bedside monitor (Drager Infinity Delta XL, Dräger Medical GmbH Lübeck, Germany). Intermittent thermodilution cardiac output (CO) measurements were performed using the pulmonary artery catheter by injecting 10 mL of normal saline into the superior vena cava at room temperature. Three consecutive injections were randomly made during the respiratory cycle. If measurements differed by >10%, the cardiac output was measured two more times and a mean was calculated after exclusion of the highest and lowest values. To avoid variation between operators, the injections were always performed by the same experienced operator. Washout curves were examined for stable baseline temperature, undisturbed rapid upstroke, and exponential decay without signs of early recirculation. Cardiac output was normalized for total body surface area to obtain the cardiac index (CI). Blood samples (2 mL per sample) were taken from the tip of pulmonary artery catheter to measure SvO_2_. The correct positioning of the catheter was confirmed by the waveform of the pressure curve, catheter length, and chest X-ray. SvO_2_ was measured (*ABL800 FLEX*,* Radiometer Medical ApS*,* 2700 Brønshøj*,* Denmark*).

The following hemodynamic parameters were calculated: arterial oxygen saturation (SaO_2_), mixed venous oxygen saturation (SvO_2_), oxygen delivery index (DO_2_I), oxygen consumption index (VO_2_I), oxygen extraction ratio (O_2_ER), central venous pressure (CVP), cardiac index (CI), left ventricular stroke work index (LVSWI), heart rate (HR), pulmonary capillary pressure (PCP), and mean arterial pressure (MAP).

#### 2.2.2. Color and Pulse Doppler Ultrasonography

All ultrasonographic examinations were performed by a single expert operator using a Philips CX50 Echocardiography (*Philips Healthcare*,* Eindhoven*,* Netherlands*) with a S5-1 Sector Array transducer for kidney's examination with the patient in supine position. After a general preliminary examination of the abdominal cavity and organs, Doppler ultrasound measurements were obtained in the interlobar arteries of the renal cortex. The ultrasound examination was considered technically adequate if the following criteria were met: (a) a clear two-dimensional longitudinal scan with definition of renal parenchyma, (b) a good color Doppler image with representation of the intrarenal vascular blood flow, and (c) at least three consecutive Doppler time-velocity spectra for each renal area (upper, middle, and lower regions). Waveforms were recorded and renal Doppler resistive index was calculated according to Planiol and Pourcelot protocol [7]. For each of the three renal areas, three Doppler measurements were taken, and the mean values were then averaged to derive an index for the whole organ in order to minimize sampling error. Pulsed wave Doppler spectrum was increased by using the lowest frequency shift range that did not cause aliasing and the wall filter was set at a low frequency (100 MHz). Values of renal Doppler resistive index > 0.70 were considered abnormal, with normal values ranging between 0.48 and 0.68 [8]. Renal venous flow was evaluated to exclude the presence of occlusion or thrombosis.

### 2.3. Statistical Analyses

Results were expressed as mean ± standard deviation or percentage. Categorical data were compared by Pearson's *χ* ^2^ test with Yates correction or Fisher's exact test when appropriate. Continuous variables were compared with Student *t*-test for unpaired data.

Relationships between RDRI and hemodynamic indexes were evaluated by Pearson's correlation coefficient. A power calculation for correlation test was performed as previously described [9]. Main hemodynamic and clinical continuous variables were entered into univariate linear regression models, in which RDRI was set as dependent variable. The variables that reached statistical significance at the univariate analysis, without violating the assumption of no multicollinearity, were then entered into a multivariate linear regression model.

A two-sided *P* value < 0.05 was assumed as statistically significant. Statistical analyses were performed using SPSS 20.0 (SPSS, Chicago, Ill), GraphPad Prism 6.00 (GraphPad Software, San Diego, CA), and the R software/environment (version 3.1.2; R Foundation for Statistical Computing, Vienna, Austria).

## 3. Results

A power calculation carried out for a linear correlation coefficient of 0.4 with type-I error probability of 0.05 returned a statistical power of 0.90 with a sample size of 61.

According to inclusion criteria, no patient had creatinine levels outside the normal range (0.50–1.20 mg/d). The creatinine mean value at admission was 1.1 ± 0.1 mg/dL. RDRI was adequately measureable from both kidneys in 56 patients and from the right kidney alone in the remaining 5 patients, because of suboptimal visualization of left kidney due to overlying bowel gas. Thus 117 measurements were obtained in the 61 patients. Eleven patients had RDRI >0.70. When subjects were grouped on the basis of this cutoff, those with RDRI >0.7 had higher heart rate, lower SaO_2_ and SvO_2_, and larger O_2_ extraction ratio (Table 2). No other hemodynamic variable was significantly different between the two groups.

**Figure 1:**
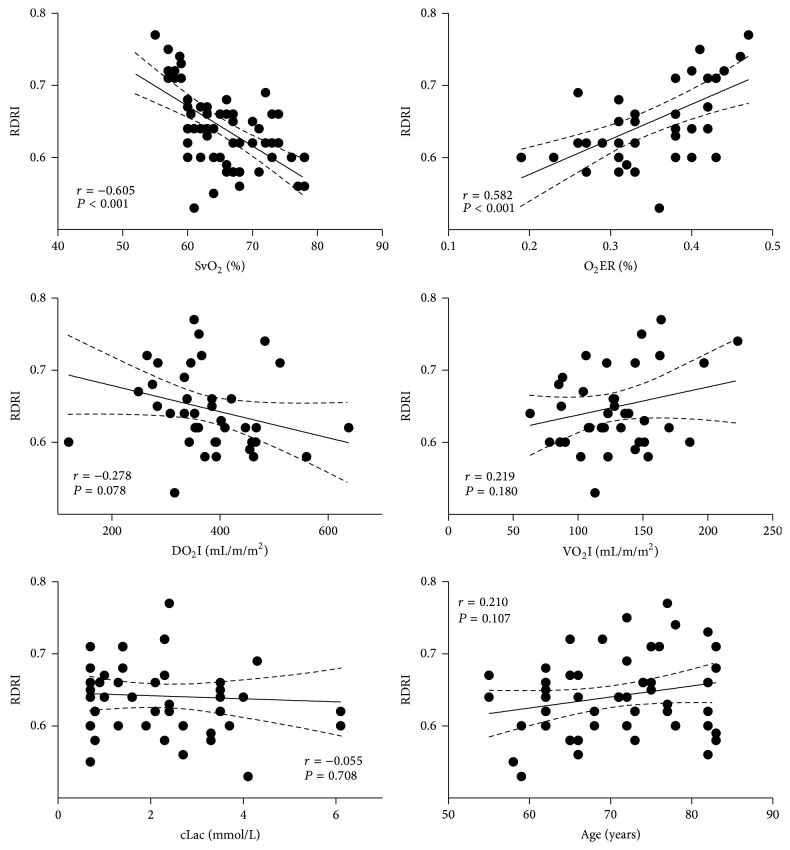
Correlations of renal Doppler resistive (RDRI) index with SvO_2_: mixed venous oxygen saturation, O_2_ER: oxygen extraction ratio [VO_2_I/DO_2_I], DO_2_I: oxygen delivery index [CI × Hb × 1.34 × SaO_2_ + 0.003 × PaO_2_], VO_2_I: oxygen consumption index [CI × Hb × 1.34  ×  SvO_2_ + 0.003 × PvO_2_], cLac: arterial lactate concentration, and age, in 61 patients admitted to the intensive care unit after elective cardiac surgery.

**Figure 2:**
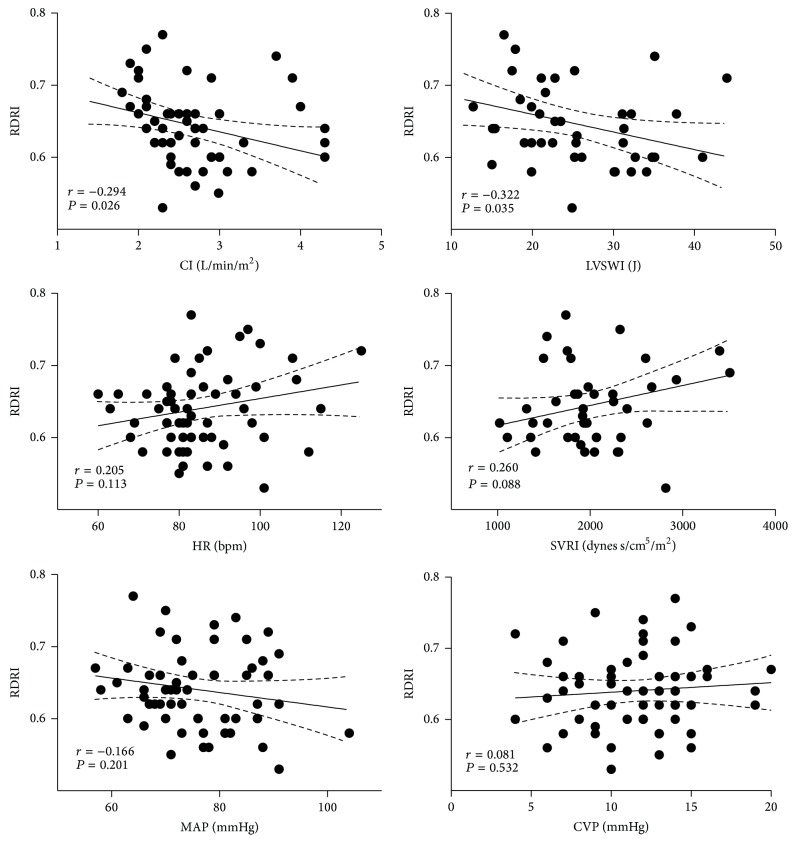
Correlations of renal Doppler resistive (RDRI) index with CI: cardiac index, LVSWI: left ventricular stroke work index [(MAP_(mmHg)_  −  PCWP_(mmHg)_)  ×  SVI_(mL)_  ×  0,0133322], HR: heart rate, SVRI: systemic vascular resistance index, MAP: mean artery pressure, and CVP: central venous pressure, in 61 patients admitted to the intensive care unit after elective cardiac surgery.

RDRI showed a significant inverse correlation with SvO_2_ (*r* = −0.605; *P* < 0.001), O_2_-ER (*r* = −0.582; *P* < 0.001), CI (*r* = −0.294; *P* = 0.0265), and LVSWI (*r* = −0.322; *P* = 0.035), but not with HR (*r* = 0.205; *P* = 0.113), CVP (*r* = 0.081; *P* = 0.532), MAP (*r* = −0.166; *P* = 0.201), PCP (*r* = 0.059; *P* = 0.650), SVRI (*r* = 0.260; *P* = 0.088), DO_2_I (*r* = −0.278; *P* = 0.078), VO_2_I (*r* = 0.219; *P* = 0.180), arterial lactate concentration (*r* = −0.055; *P* = 0.708), and age (*r* = 0.210; *P* = 0.107) (Figures 1 and 2).

Potential predictive hemodynamic parameters for RDRI were entered into univariate linear regression models. A statistical significance was found for LVSWI (*P* = 0.035), CI (*P* = 0.029), O_2_-ER (*P* < 0.001), and SvO_2_ (*P* < 0.001) (Table 3). O_2_-ER and LVSWI were not included in the multivariate analysis because they were highly correlated with SvO_2_ and CI, respectively. The multivariate regression model including SvO_2_ and CI as independent variables yielded an overall *R* ^2^ of 0.390 (*P* < 0.045), with SvO_2_ being the major contributor (*P* < 0.001). Even when age was forced in the multivariate analysis the overall significance of the model did not change (*R* ^2^ = 0.42; *P* < 0.045), confirming SvO_2_ as the major contributor (Table 3).

Patients with SvO_2_ < 55% had a RDRI significantly higher than those with SvO_2_ ≥ 55% (0.73 ± 0.04 versus 0.63 ± 0.04; *P* < 0.001).

The evolution of patients with high RDRI did not differ from that observed in patients with normal values in terms of ICU length of stay (8 ± 6 versus 7 ± 6 days; *P* = 0.763), hospital length of stay (18 ± 10 versus 17 ± 10 days; *P* = 0.857), ICU mortality (0 versus 4 patients; *P* = 0.459), number of red blood cell transfusion requirements (4 ± 4 versus 5 ± 4; *P* = 0.447), time of mechanical ventilation (108 ± 126 versus 84 ± 132 h; *P* = 0.604), intra-aortic counter pulsation (2 versus 4; *P* = 0.247), and number of patients requiring hemodialysis (0 versus 1; *P* = 0.831).

## 4. Discussion

High RDRI values were previously described to be a reliable predictor of occult or incipient hemorrhagic shock in hemodynamically stable polytrauma patients, with low arterial lactate levels and normal hemoglobin at admission [4]. Moreover, a significant correlation between RDRI and arterial standard base excess, as a marker of tissue hypoxia, was consistently found in polytrauma patients [5]. Also in patients with acute lung injury, RDRI has been described to increase during short-term low-FiO_2_ titration, thus providing evidence of substantial renal effects due to hypoxemia [6].

The main finding of the present study is that RDRI was significantly correlated with SvO_2_ and cardiac index, independent of the presence of other hemodynamic abnormalities. This supports the hypothesis of an early and significant response of the renal vasculature to an even mild oxygen supply and demand mismatch. To the best of our knowledge, this is the first study showing a correlation between RDRI and invasive hemodynamic measurements, particularly SvO_2_. We observed a negative correlation between RDRI and SvO_2_ but not an inverse correlation between RDRI and mean arterial pressure. This finding suggests that the renal vasoconstrictor response is likely to be modulated not only by a reduction in effective circulating volume, but also by mechanisms depending on oxygen supply and demand mismatch, as expressed by SvO_2_. This pathophysiologic response may be triggered even in the presence of normal arterial oxygenation [10].

One possible explanation is that kidney is highly sensitive to ischemic injury, due to its complex microvascular structure coupled with high metabolic needs. Under normal steady-state conditions, the oxygen supply to the renal parenchyma is largely exceeding its oxygen demand [10–20]. However, under pathological conditions the delicate balance between oxygen supply and demand is commonly overturned. In this context, cellular hypoxia develops in response to the decreased availability of oxygen due to inadequate convective delivery from the microcirculation.

It has been previously shown that resistance index can exceed the threshold of 0.70 in elderly subjects without renal insufficiency [21, 22], due to age-related changes in vascular compliance, atherosclerosis, or presence of arterial hypertension-related vascular damage [23, 24]. In our study, age was not identified as an independent determinant of RDRI in both uni- and multivariate regression analysis probably due to the narrow age range of patients.

Most patients admitted to ICU after cardiac surgery need support by vasoactive drugs, which can modify the vascular tone of renal arteries, thus probably affecting RDRI measurements. In the present study the use of vasoactive drugs did not significantly differ between patients with RDRI above or below 0.7, as documented by the vasoactive-inotropic score (VIS) calculated for each patient at each measurement.

This study has some limitations. First, our critically ill patients were sedated and mechanically ventilated with standardized ventilation after cardiac surgery, and thus results cannot be extrapolated to either spontaneously breathing patients or other critical conditions requiring protective mechanical ventilation. Second, patients with increased creatinine levels were excluded from the study. Although this makes the results not applicable to patients with acute or chronic renal failure, it allowed us to avoid confounding factors influencing renal vasoconstriction. Third, the age and the use of vasoactive drugs were quite similar among patients, which may have prevented finding significant correlations. Nevertheless, the narrow range of age and the similar use of vasoactive drugs in all patients allowed us to describe the response of RDRI to tissue hypoxia independently of other confounding factors. Fourth, the study was not potentiated to find differences in terms of clinical outcomes.

In conclusion, the results of the present study suggest that RDRI is sensitive to oxygen supply and changes in SvO_2_, supposedly reflecting a vascular response to hypoxia.

## Figures and Tables

**Table 1:** Baseline characteristics of the population at inclusion.

Baseline characteristics at inclusion	All patients	RDRI > 0.7	RDRI ≤ 0.7	*P*
Age, years	70 ± 8	75 ± 6	70 ± 8	0.510
Sex, m/f	52/9	9/2	43/7	0.616
Simplified acute physiology score II	27 ± 11	27 ± 11	27 ± 11	0.940
EuroSCORE	6 ± 3	7 ± 2	6 ± 3	0.445
Ejection fraction, % (before surgery)	42 ± 13	50 ± 11	41 ± 12	0.580
Creatinine, mg/dL (before surgery)	1.1 ± 0.1	0.9 ± 0.2	1 ± 0.2	0.188
Vasoactive inotropic score	23 ± 15	22 ± 11	24 ± 16	0.909

**Table 2:** Hemodynamic data and oxygenation parameters.

	All	RDRI > 0.7	RDRI ≤ 0.7	*P*
Heart rate (bpm)	86 ± 12	94 ± 13	84 ± 12	0.028
MAP (mmHg)	76 ± 10	77 ± 10	76 ± 10	0.708
MPAP (mmHg)	24 ± 5	24 ± 4	24 ± 5	0.601
CI (L/min/m^2^)	2.7 ± 0.6	2.5 ± 0.8	2.7 ± 0.6	0.466
LVSWI (Joule)	25 ± 8	24 ± 10	26 ± 7	0.412
CVP (mmHg)	12 ± 3	11 ± 4	12 ± 3	0.864
PCP (mmHg)	14 ± 5	14 ± 5	15 ± 5	0.523
SVRI (dynes s/cm^5^/m^2^)	2044 ± 558	2191 ± 687	2001 ± 517	0.434
PVRI (dynes s/cm^5^/m^2^)	300 ± 160	373 ± 205	277 ± 140	0.189
Hemoglobin (g/dL)	11 ± 2	11 ± 2	11 ± 2	0.539
Lactate concentration (mmol/L)	1.5 ± 1	1.5 ± 0.4	1.5 ± 0.9	0.925
SaO_2_ (%)	98 ± 2	97 ± 2	99 ± 2	0.033
SvO_2_ (%)	66 ± 6	57 ± 2	67 ± 6	<0.001
ΔCO_2_ (mmHg)	5 ± 2	6 ± 2	5 ± 3	0.492
DO_2_I (mL/m/m^2^)	378 ± 93	355 ± 88	385 ± 94	0.361
VO_2_I (mL/m/m^2^)	128 ± 33	146 ± 40	122 ± 29	0.110
O_2_ER (%)	35 ± 0.07	41 ± 0.04	32 ± 0.06	<0.001
RDRI	0.64 ± 0.06	0.74 ± 0.03	0.62 ± 0.04	<0.001

MAP: mean artery pressure; MPAP: mean pulmonary artery pressure; CI: cardiac index; LVSWI: left ventricular stroke work index [(MAP_(mmHg)_  −  PCWP_(mmHg)_) ×  SVI_(mL)_  × 0,0133322]; CVP: central venous pressure; PCP: pulmonary capillary pressure; SVRI: systemic vascular resistance index; PVRI: pulmonary vascular resistance index; SaO_2_: arterial oxygen saturation; SvO_2_: mixed venous oxygen saturation; ΔCO_2_: venoarterial CO_2 _gradient; DO_2_I: oxygen delivery index [CI × Hb × 1.34 × SaO_2_ + 0.003 × PaO_2_]; VO_2_I: oxygen consumption index [CI × Hb × 1.34 × SvO_2_ + 0.003 × PvO_2_]; O_2_ER: oxygen extraction ratio [VO_2_I/DO_2_I]; RDRI: renal Doppler resistive index; Hb: hemoglobin (gr/mL); PaO_2_: partial pressure of arterial oxygen (mmHg); PvO_2_: partial pressure of mixed venous oxygen (mmHg).

**Table 3:** Univariate and multivariate linear regression analysis of potential predictive hemodynamic parameters for renal Doppler resistive index.

Parameter	Univariate analysis			Multivariate analysis			Multivariate analysis		
	*β*	SE	*P*	*β*	SE	*P*	*β*	SE	*P*
Lactate	0.041	0.014	0.840			Not entered			
PCP	−0.059	0.001	0.650			Not entered			
CVP	0.082	0.002	0.531			Not entered			
MAP	−0.167	0.001	0.199			Not entered			
Heart rate	0.204	0.001	0.115			Not entered			
Age	0.211	0.001	0.106			Not entered	0.179	0.001	0.110
VO_2_I	0.223	0.001	0.178			Not entered			
SVRI	0.259	0.001	0.090			Not entered			
DO_2_I	−0.277	0.001	0.079			Not entered			
LVSWI^*∗*^	−0.321	0.001	0.036			Not entered			
Cardiac index	−0.293	0.012	0.027	−0.226	0.010	0.039	−0.181	0.010	0.107
O_2_ER^*∗*^	0.581	0.114	<0.001			Not entered			
SvO_2_	−0.604	0.001	<0.001	−0.555	0.001	<0.001	−0.573	0.001	<0.001

*β*: regression coefficient; SE: standard error. ^*∗*^Variables excluded from the multivariate analysis because of the violation of assumption of no multicollinearity.
